# Physical exercise and goal attainment after shunt surgery in idiopathic normal pressure hydrocephalus: a randomised clinical trial

**DOI:** 10.1186/s12987-021-00287-8

**Published:** 2021-11-22

**Authors:** Johanna Rydja, Lena Kollén, Per Hellström, Katarina Owen, Åsa Lundgren Nilsson, Carsten Wikkelsø, Mats Tullberg, Fredrik Lundin

**Affiliations:** 1 Department of Activity and Health, and Department of Biomedical and Clinical Sciences, Linköping University, Linköping University Hospital, 581 85 Linköping, Sweden; 2 Institute of Neuroscience and Physiology, Sahlgrenska Academy, University of Gothenburg, Gothenburg, Sweden; 3 Department of Activity and Health, and Department of Health, Medicine and Caring Sciences, Linköping University, Linköping, Sweden; 4 Department of Neurology, and Department of Biomedical and Clinical Sciences, Linköping University, Linköping, Sweden

**Keywords:** Idiopathic normal pressure hydrocephalus, Neurosurgical procedures, Rehabilitation, Exercise

## Abstract

**Background:**

Rehabilitation in iNPH is suggested to be an important factor to improve patients’ functions but there are lack of clinical trials evaluating the effect of rehabilitation interventions after shunt surgery in iNPH. The objective of this study was to evaluate the effect of a physical exercise programme and goal attainment for patients with idiopathic normal pressure hydrocephalus (iNPH) after surgery compared to a control group.

**Methods:**

This was a dual centre randomised controlled trial with assessor blinding, intention-to-treat (ITT) and per protocol (PP) analysis. Individuals diagnosed with iNPH scheduled to undergo shunt surgery at the Linköping University Hospital in Linköping and Sahlgrenska University Hospital in Gothenburg, Sweden were consecutively eligible for inclusion. Inclusion was conducted between January 2016 and June 2018. The patients were randomised 1:1 using sequentially numbered sealed envelopes to receive either written exercise information (control group) or written information and an additional supervised high-intensity, functional exercise programme (HIFE) executed twice weekly over 12 weeks (exercise group). Preoperatively, the patients set individual goals. The primary outcome was change from baseline in the total iNPH scale score at the post-intervention follow-up. Secondary outcomes were goal attainment, and change in the separate scores of gait, balance, neuropsychology and continence and in the total score after 6 months.

**Results:**

In total, 127 participants were randomised to the exercise group (n = 62) and to the control group (n = 65). In the ITT population (exercise group, n = 50; control group, n = 59), there were no between-group differences in the primary outcome, but the attrition rate in the exercise group was high. The exercise group improved more than the control group in the balance domain scores after 6 months. Post-intervention, the PP exercise population achieved their set goals to a greater extent than the controls.

**Conclusions:**

An additional effect of the 12-week HIFE-programme on the overall improvement according to the iNPH-scale after shunt surgery in iNPH was not shown. This could be due to high attrition rate. However, the long-term effect on balance and higher goal achievement indicate beneficial influences of supervised physical exercise.

*Trial registration* clinicaltrials.gov, NCT02659111. Registered 20 January 2016, https://clinicaltrials.gov/ct2/show/NCT02659111

**Supplementary Information:**

The online version contains supplementary material available at 10.1186/s12987-021-00287-8.

## Introduction

The core clinical symptoms in idiopathic normal pressure hydrocephalus (iNPH) are impaired gait in combination with a disturbed balance [1–3] often in conjunction with cognitive impairment and incontinence [4]. The iNPH scale is a calibrated, norm-based grading scale assessing postoperative outcome in iNPH and previously used in the European multi-centre study [5]. The scale covers four symptom domains and a total score is calculated [6]. Approximately 80% of the patients are reported to benefit from shunt surgery [5, 7]. However, patients with iNPH are more sedentary in comparison with healthy controls of the same age. Despite the alleviation of symptoms, the patients’ overall physical activity does not increase after surgery [8].

In Parkinson’s disease with a symptomatology similar to iNPH, physical exercise is an essential part of the treatment [9, 10]. Goal-oriented aerobic exercise has been suggested to promote neuroplasticity, motor and cognitive improvements [11]. Exercise programmes lasting for more than 12 weeks have long-term effects [12]. Rehabilitation in iNPH is emphasised to be an important factor to improve patients’ functions [13, 14]. A recently published non-randomised clinical trial evaluating home exercise in iNPH concluded that home exercise was easy to implement and accepted by the patients [15]. However, no randomised clinical trial has explored the effectiveness of rehabilitation in iNPH.

The aim of this study was to evaluate the effect on outcome and goal achievement of an added physical exercise programme for patients with iNPH after shunt surgery, compared to a control group of iNPH patients. We hypothesised that the high-intensity functional exercise programme (HIFE) [16] would have positive effects on the main symptom domains in iNPH evaluated with the iNPH scale [6].

## Methods

### Participants

Consecutive patients diagnosed with iNPH according to the international guidelines [4] and waiting for shunt surgery were eligible for inclusion. Exclusion criteria were Mini Mental State Examination (MMSE) [17] < 16, inability to walk with or without walking aids for > 10 m or suffering from other diseases making intensive exercise impossible. After the decision on surgery, eligible patients were included and randomised. All patients had a ventriculo-peritoneal shunt with either a Strata™ Valve, n = 71 (Medtronic PS Medical, Santa Barbara, USA), a Codman® Medos® Hakim Valve, n = 49, or a Codman® Certas® Plus, n = 7 (Integra LifeSciences Corporation). Participants were recruited from two Swedish Hydrocephalus centres at Linköping University hospital (Linköping) and Sahlgrenska University hospital (Gothenburg).

The study conforms with Declaration of Helsinki guidelines and the medical ethical committee of Linköping approved the study, Approval Number: 2015/250-31. The study protocol was published in advance at clinicaltrials.gov, Id: NCT02659111. Eligible patients received oral and written information and gave written informed consent of acceptance.

### Randomisation and blinding

One person at each centre, neither participating in the assessments nor in the data analysis, randomly assigned eligible patients 1:1 using sequentially sealed envelopes, to the added intervention (exercise group) or to usual care (control group) without stratification. The sealed envelopes were randomly computer generated and the sequential management ensured the randomisation. The assessors at the two centres were blinded after assignment to the interventions and in order to keep the randomisation, the patients were asked to keep their intervention confidential. The randomisation key was opened after the last six-month follow-up.

### Intervention

In the usual care routine both groups received the same written and oral information about physical activity and eight standardised low-intensity home-based exercises (Additional File 1). All participants in both groups set individual goals, comprising concrete descriptions of what they were hoping to accomplish following treatment, such as being able to walk a specified distance, e.g. to the supermarket. The goals were set with a physiotherapist (in Gothenburg) or an occupational therapist (in Linköping), in conjunction with hospital visits for preoperative assessments.

Following surgery, the exercise group underwent an individual high-intensity functional exercise programme, the HIFE programme^(^™^)^ [16, 18] for one hour, twice a week, for 12 weeks. The HIFE programme is a battery of functional weight-bearing exercises focusing on balance and gait. The exercises are based on everyday movements, e.g. standing up from a sitting position or walking over obstacles performed at high physical intensity. In order to increase the effort during progress, a weight belt is used. The programme is described in detail elsewhere [18]. After the 12-week intervention period the patients were motivated to continue with the physical activity.

The HIFE programme was performed under the supervision of local physiotherapists in outpatient clinics. The supervising physiotherapists discussed the patients’ goal settings and encouraged activity. A coordinator from one of the centres contacted the patient and the local physiotherapist to initiate the intervention. A standardised exercise manual [18], a weight belt and a protocol were sent to the physiotherapist and the coordinator maintained contact with the physiotherapist during the intervention.

### Outcome measures

The follow-up occasions were after the 12-week intervention and 6 months postoperatively. The iNPH scale introduced by Hellström et al. [6] consists of four domains; gait, balance, neuropsychology and continence. Different ordinal scales and continuous measures are used to quantify symptoms in each domain and raw scores are converted to a min–max range from 0 to 100 for all domains separately as well as for the total scale (Additional File 2). The primary efficacy variable was change in the total iNPH scale score from baseline to the follow-up at the end of intervention. Secondary efficacy variables were changes in the four separate domain scores and goal attainment at the end of the intervention and at the six-month follow-up and change in the total iNPH scale score at the six-month follow-up.

To assess goal attainment, we used a modified version of the goal attainment scaling (GAS) [19]. In the analysis we created a simplified dichotomous scale: 0, 1 or 2 = goal is achieved or exceeded, − 1 or − 2 = goal is not achieved. The patients answered the question: *“What would you like to achieve after the shunt surgery?”* To make the desired goal evaluable, the first question was followed by questions to specify the goal e.g. walking distance and/or frequency.

In the preparing study period, the research group met repeatedly in order to strengthen the reliability of the assessments, and the occupational therapist and the physiotherapists from the two centres co-evaluated a pilot patient.

### Statistical analysis

A sample size analysis according to the score of the total iNPH scale was performed prior to the study start. We assumed that we would find a difference in improvement of five points, with an expected improvement in the control group of 16 points and a hypothesised improvement in the exercise group of 21 points, with the same SD in both groups of 10 points and a significance level of 5%. With 80% power and a two-sided T-test/Fisher’s non-parametric permutation test, 63 participants were needed in each group.

According to the statistical analysis plan, primary and secondary analyses were carried out with the intention-to-treat (ITT) population defined as all randomly assigned patients with at least one follow-up assessment. Missing data were not replaced. A complementary analysis was performed on the per protocol (PP) population. To be included in the PP population the participants in both groups must have completed all the follow-up assessments and the participants in the exercise group attended at least 18 of the 24 intervention sessions within 4 months postoperatively.

For comparison within groups, Fisher’s non-parametric permutation test for matched pairs was used. The confidence interval for dichotomous variables was the unconditional exact confidence limits. If no exact limits could be computed, the asymptotic Wald confidence limits with continuity correction were calculated instead. The confidence interval for the mean difference between groups was based on Fisher’s non-parametric permutation test. For comparison between groups, Fisher’s non-parametric permutation test was used for continuous variables, Fisher’s exact test for dichotomous variables, and a Chi-square test for non-ordered categorical variables. The main results are presented as the mean difference between the two randomised groups with 95% CI. All statistical tests were two-tailed and conducted at the 0.05 significance level.

For variables with significant differences at baseline, a mixed model was used with the explanatory variables; baseline variable, treatment group and the interaction between them. For each model, the F test was used to test the significance of interaction between a baseline variable and treatment group.

Exploratory interaction analyses between treatment group and baseline variables were performed for primary efficacy variables and for selected secondary variables. Baseline variables with interaction *p* < 0.10 were further investigated with sub-group analyses. A professional statistician conducted the statistical analyses with SAS® v9.2 (Cary, NC).

## Results

### Participant’s flow and dropouts

Participants were recruited from January 2016 until June 2018 and the last six-month follow-up was completed in May 2019. The inclusion stopped when the calculated sample size was achieved and 131 patients were allocated for randomisation. Four patients were excluded because of incorrect randomisation: two were diagnosed with secondary NPH and two did not undergo surgery. Of the remaining 127 participants, 62 were randomised to the exercise group and 65 to the control group.

Fifty individuals in the exercise group and 59 in the control group were available for the ITT follow-up analysis at the end of the intervention. Twelve participants in the exercise group were lost to the follow-up assessment; two died due to a subdural haematoma, one moved abroad, four had subdural haematomas treated with an elevated valve opening pressure setting, one had a distal shunt catheter dysfunction, one had a shunt infection and three participants withdrew their consent. In the control group, six participants were lost at the end of intervention follow-up; one died due to cardiac arrest approximately 3 months after surgery, four had subdural haematomas treated with an elevated valve opening pressure, and one had an intra-cerebral haemorrhage.

Twenty-eight participants in the exercise group and 58 in the control group were available for the PP analysis at the end of the intervention. In addition to the dropouts in the ITT population, four participants declined to participate in the intervention or withdrew consent, nine could not start the intervention due to logistic reasons and nine participants had fewer than 18 intervention sessions. In the control group, one participant was lost due to headache caused by shunt over-drainage.

**Fig. 1:**
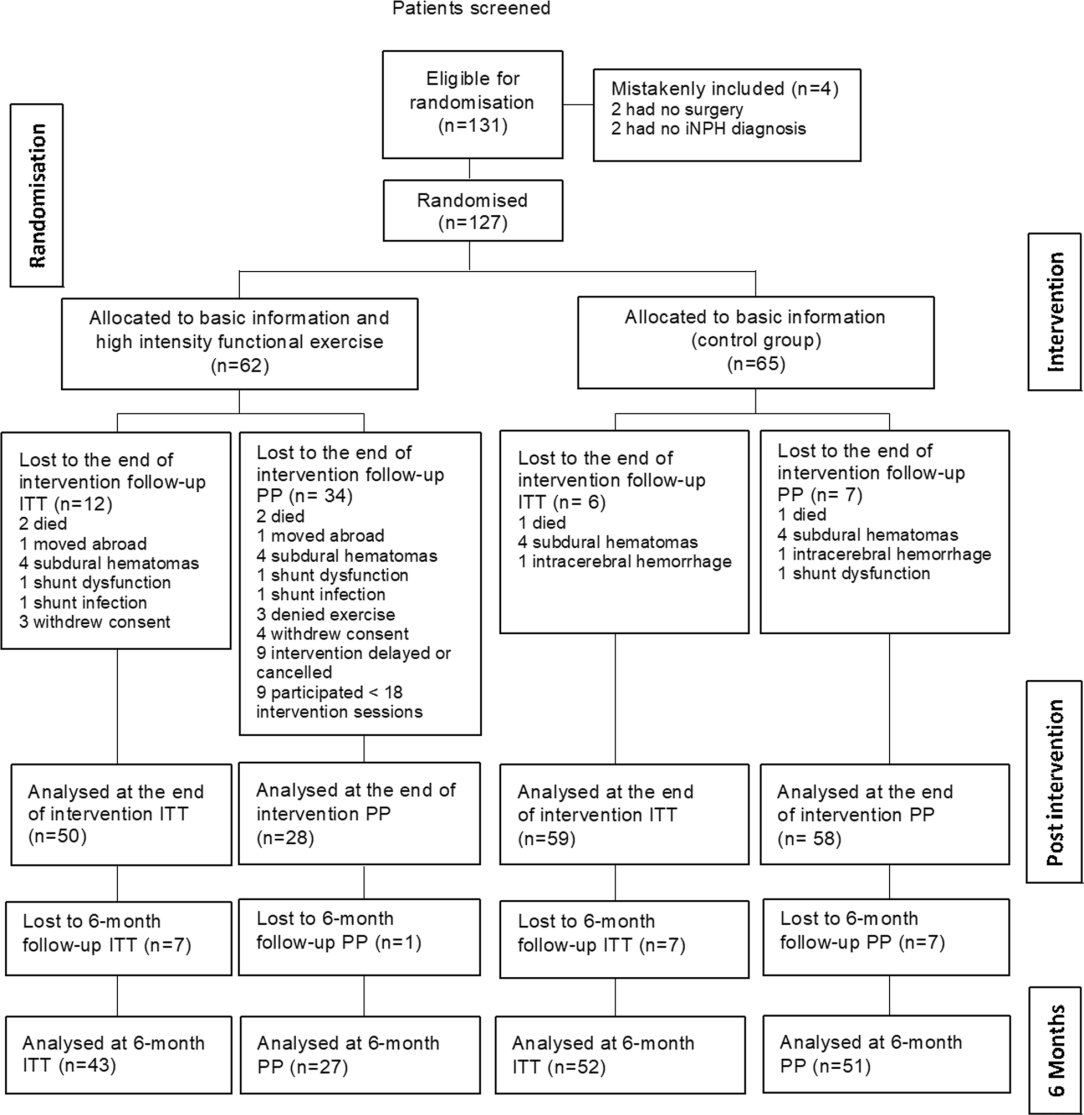
CONSORT flowchart of included patients in the intention-to-treat (ITT) and per protocol (PP) analyses. ITT was defined as all randomised participants with at least one follow-up assessment. PP was defined as all randomised participants with complete follow-up assessments and in the exercise group at least 18 exercise sessions

At the six-month follow-up, 43 participants in the exercise group and 51 in the control group were available for the ITT analysis and 27 participants in the exercise group and 50 in the control group for the PP analysis (Fig. 1). If it was necessary to confirm a working shunt at the follow-up, radiological control followed by either CSF-dynamic testing or radionuclide shuntography was performed.

### Background characteristics

The background characteristics and medical history of the ITT population are presented in Table 1. All characteristics were balanced except for age, which was higher in the control group. The same unbalanced age was seen in the PP population. There were no interaction effects of age on the primary or the secondary variables in the iNPH scale.

**Table 1:** Baseline characteristics of the ITT population in the exercise group and the control group

	Exercise group (n = 50)	Control group (n = 59)	*p*-value
Female/male (female %)	20/30 (40.0%)	24/35 (40.7%)	1.00
Age (mean, SD)	72.1 (5.7)	75.0 (7.0)	**0.02**
MMSE (mean, SD)	26.2 (3.1)	25.6 (3.0)	0.28
BMI (mean, SD)	27.9 (4.3)	27.0 (4.0)	0.57
Smoking, Yes/No (Yes %)	6/44 (12.0%)	6/50 (10.7%)	1.00
Diabetes, Yes/No (Yes %)	20/30 (40.0%)	14/45 (23.7%)	0.11
Hypertension, Yes/No (Yes %)	33/17 (66.0%)	38/21 (64.4%)	1.00
Stroke, Yes/No (Yes %)	4/46 (8.0%)	5/54 (8.5%)	1.00
Cardiovascular disease, Yes/No (Yes %)	7/43 (14.0%)	12/47 (20.3%)	0.54
Atrial fibrillation, Yes/No (Yes %)	5/45 (10.0%)	6/53 (10.2%)	1.00

Values are presented as proportions (%) or mean (SD)

*ITT* Intention-to-treat, *SD* standard deviation, *MMSE* mini mental state examination (0–30 points), *BMI* body mass index

Bold value indicate significance of p value ≤ 0.05

### Effects of intervention

No harms or negative effects because of the intervention were reported.

### Change in iNPH scale score

**Fig. 2:**
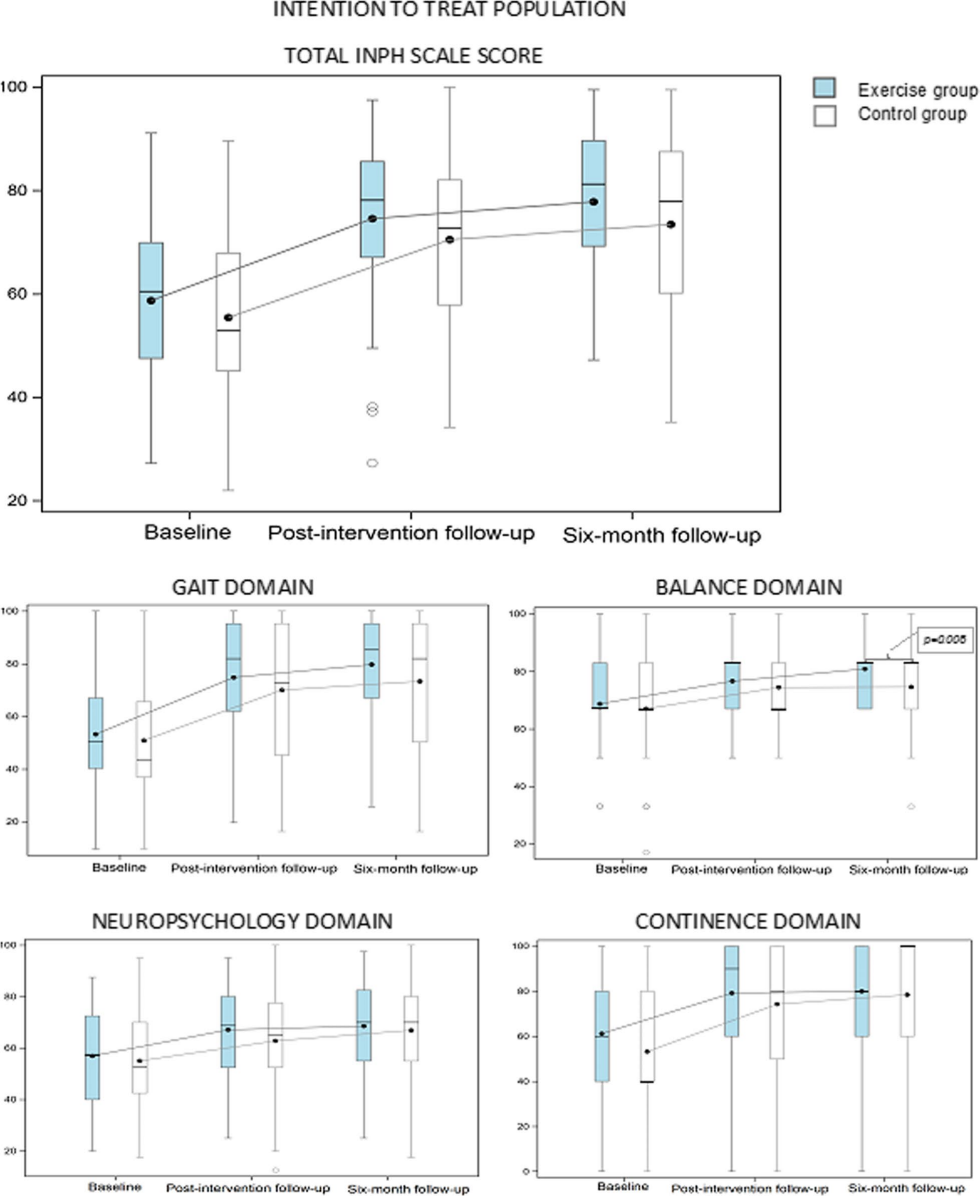
The total iNPH scale score and the separate domain scores (0–100) at baseline, at the post-intervention follow-up and at the 6-month follow-up in the intention-to-treat population (exercise group n = 50; control group n = 59). Line in box is median and the marker shows the mean. All values at the post-intervention follow-up and the six-month follow-up in both exercise group and control group are significantly changed from baseline (*p* ≤ 0.05). Significant differences between the groups are presented with *p*-value in the figure

At the post-intervention follow-up, 88/127 patients (80.7%) improved ≥ 5 points on the total iNPH scale score. Compared to baseline, both the exercise group and the control group improved at the post-intervention follow-up and after 6 months. There were no between-group differences regarding change in the total iNPH scale scores from baseline at any of the follow-up sessions. In the secondary ITT analysis of each iNPH scale domain, the exercise group had higher balance domain scores than the control group at 6 months. For all other domains there were no between-group differences, both the exercise group and the control group significantly improved in all separate domains compared to baseline at the two follow-up sessions (Fig. 2, Additional File 3).

In an exploratory interaction analysis, sex appeared to be an interacting factor and a sub-group analysis by sex was conducted. At baseline, females had lower gait domain scores than males but without significant differences (females n = 53; median 43.7, min–max 9.7–100; mean 46.0, SD 21.5 vs males n = 74, median 54.7, min–max 9.7–100; mean 55.6, SD 24.5); *p* = 0.06. At the post-intervention follow-up females in the exercise group had greater improvements in the gait domain scores from baseline (n = 20; median 25, min–max 0–54.7; mean 24.7, SD 13.6) compared to females in the control group (n = 24; median 10.8, min–max – 10 to 48.3; mean 14.6, SD 15.4), *p* = 0.026; mean 10.2 (95% CI 1.3; 19.3).

**Fig. 3:**
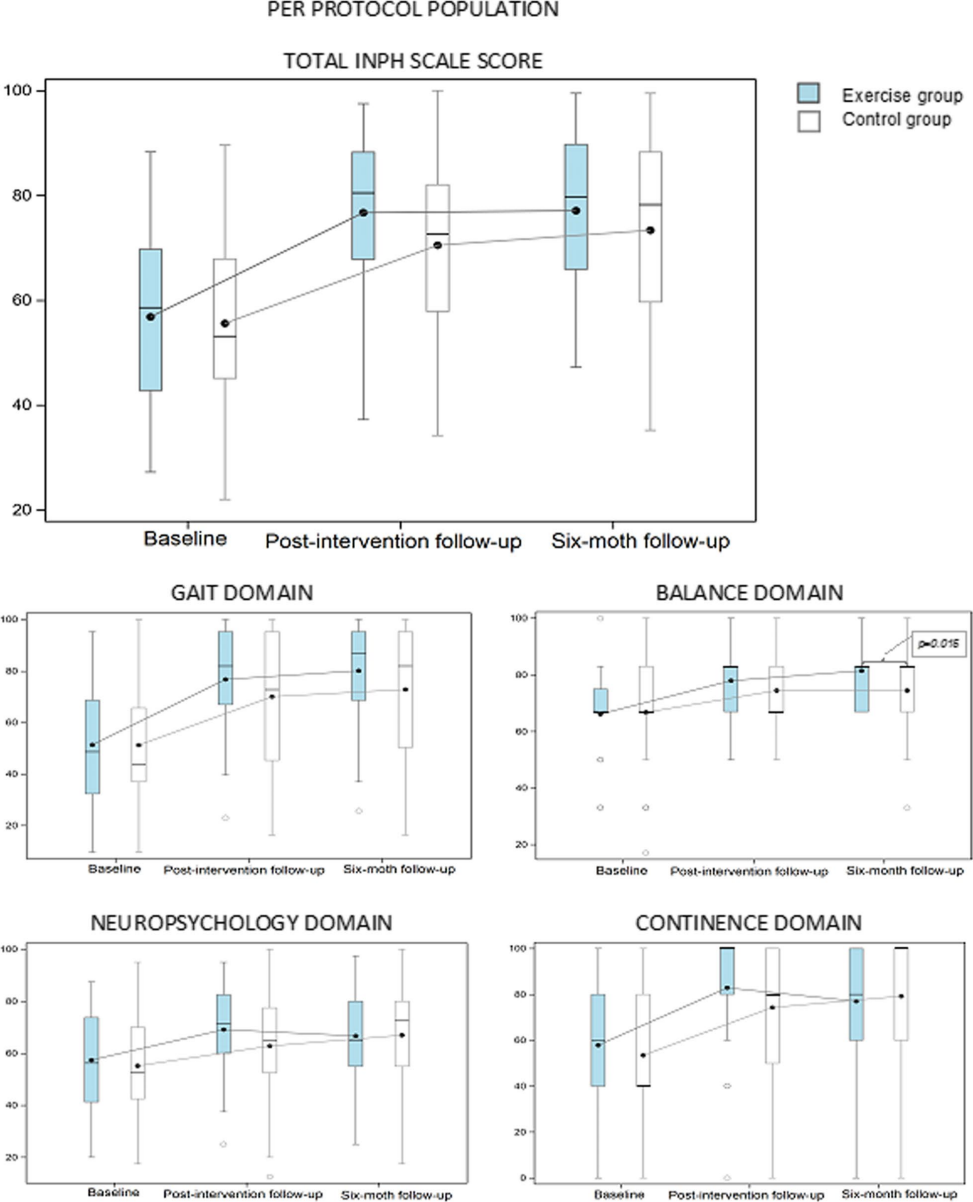
The total iNPH scale score and the separate domain scores (0–100) at baseline, at the post-intervention follow-up and at the 6-month follow-up in the per protocol population (exercise group n = 28; control group n = 58). Line in box is median and the marker shows the mean. All values at the post-intervention follow-up and at the six-month follow-up in both exercise group and control group are significantly changed from baseline (*p* ≤ 0.05). Significant differences between the groups are presented with *p*-value in the figure

Similar results for the total iNPH scale score were seen in the PP population as in the ITT population. For the secondary outcome measures there were no between-group differences in changes from baseline for any of the separate iNPH scale domains at the post-intervention follow-up. After 6 months, the exercise group had improved more than the control group in the balance domain scores compared to baseline but showed similar improvements in the other domains (Fig. 3, Additional File 4).

### Intervention effect on GAS

**Fig. 4:**
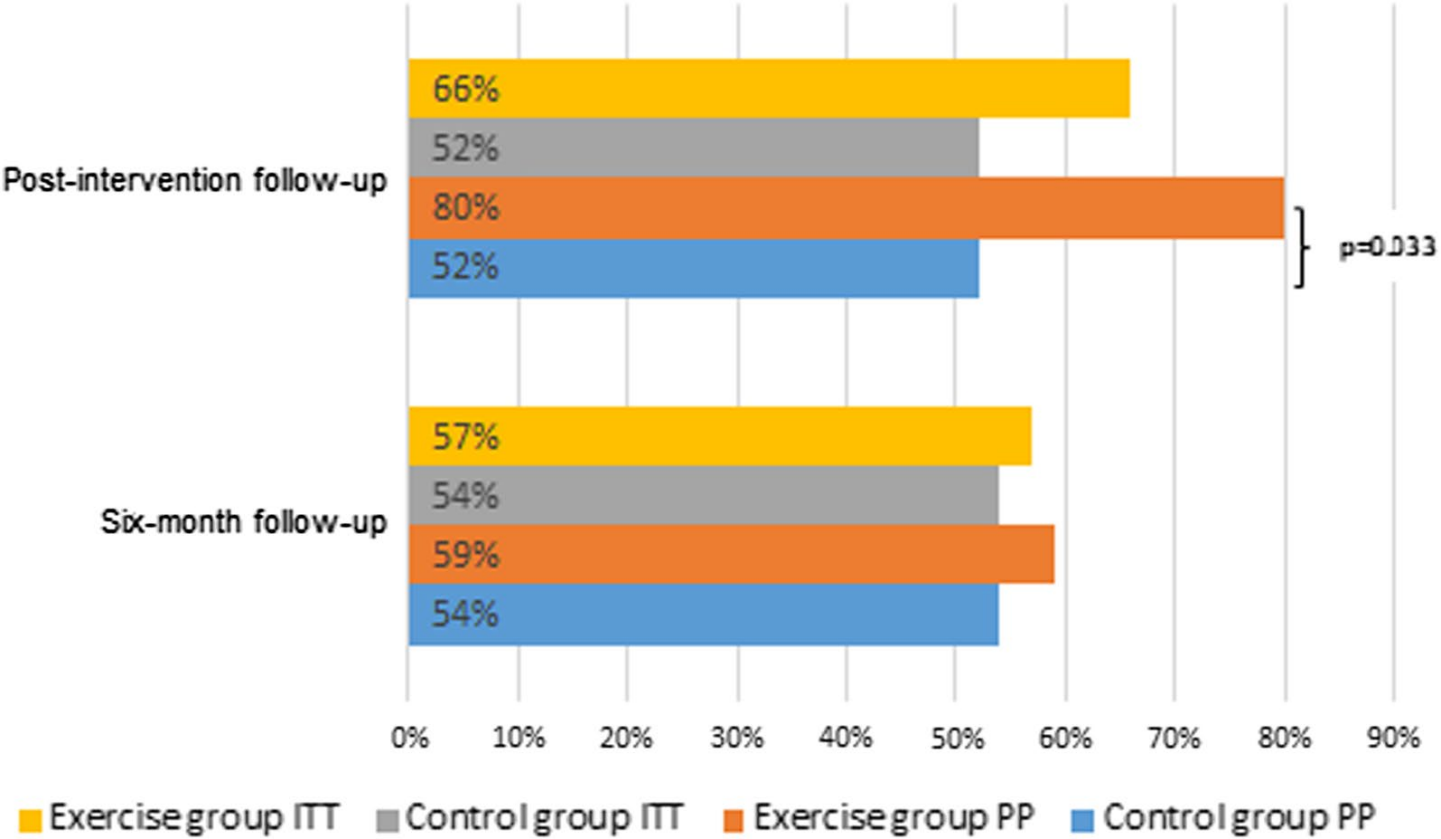
Participants with achieved goals for ITT and PP populations post-intervention and 6 months postoperatively. *ITT* intention-to-treat, *PP* per protocol. Values are presented as proportions (%). Significant difference between exercise group and control group is presented with p-value in the figure. Post-intervention follow-up (exercise group ITT n = 44, control group ITT n = 50; exercise group PP n = 25, control group PP n = 50). Six-month follow-up (exercise group ITT n = 46, control group ITT n = 50; exercise group PP n = 27, control group PP n = 50

In the ITT population, the proportions of individuals with achieved goals were equal in the exercise group compared to the control group at both the post-intervention follow-up (29 of 50, 65.9% versus 26 of 59, 52.0%; *p* = 0.25) and at the six-month follow-up (26 of 50, 56.5% versus 27 of 59, 54.0%; *p* = 0.97). In the PP population the proportions of individuals with achieved goals at the post-intervention follow-up favoured the exercise group (20 of 28, 80.0% versus 26 of 58, 52.0%; *p* = 0.033). After 6 months, the between-group difference had disappeared and the proportions of individuals with achieved goals were similar (16 of 28, 59.3% versus 27 of 58, 54.0%; *p* = 0.84), (Fig. 4).

## Discussion

This randomised clinical trial evaluates the effects of physical exercise in iNPH. Our primary hypothesis that the exercise group would show greater postoperative improvement in the total iNPH scale score than the control group was not confirmed. Secondary outcome analyses showed: (1) after 6 months, the exercise group had improved more in the balance domain scores in both ITT and PP analyses; (2) at the post-intervention follow-up, the exercise PP population achieved their goals to a greater extent.

Even if we could not show an overall effect, for reasons discussed in the limitation section, the improvement in balance after 6 months is notable. The scientific evidence of the effect of balance training in iNPH is limited. In Parkinson’s disease, different interventions improve balance and reduce the number of falls up to 12 months after the end of the interventions [12]. The HIFE programme has previously been evaluated for balance with the Berg Balance Scale as an outcome measure in a population with dementia in a nursing home. In that study there was no between-group difference compared to a low-intensive control intervention at the post-intervention follow-up but long-term effects were found with between-group differences favouring the exercise group after 6 months, [20] similar to the results from our study. Gait speed was improved in the intervention group, both at the post-intervention follow-up and at the long-term follow-up [20].

We found no significant between-group differences in the gait domain scores either in the ITT or in the PP population. However, in the PP population at the six-month follow-up, the exercise group showed a larger change from baseline than the control group at trend level. Difference between groups: mean 8.1 (95% CI − 1.3; 17.5), *p* = 0.092).

Both the ITT exercise group and the control group increased their total iNPH scale scores from the post-intervention follow-up to the six-month follow-up. Although not significant this increase may indicate a continuing improvement over time after shunt surgery and that longer follow-up periods can be valuable when evaluating outcome in iNPH patients.

The sub-group populations stratified by sex are small, which makes the gender differences in outcome less robust. However, older females are generally less physically active than males [21]. In our study the females had lower physical function at baseline (lower iNPH scale score) even if the sex differences were not significant. This may have contributed to the more pronounced intervention effect on gait among the females. However, our findings generate important questions, e.g. whether females have a lower level of function before surgery, which has been previously reported [5] or whether females wait longer with symptoms before seeking medical advice, which delays surgery, and finally whether there are any differences in rehabilitation effects.

The higher goal attainment at the post-intervention follow-up in the PP population exercise group indicates that the HIFE intervention in combination with the supervision may have influenced the goal achievements. The lack of difference in goal attainment between the exercise group and the control group at the six-month follow-up also supports this notion. Goal setting could be managed by people with mild to moderate dementia, especially when using a structured approach [22]. Impaired memory and executive difficulties in iNPH improve after shunt surgery but are still not at the same level as in healthy individuals of the same age [23]. The cognitive decline can affect the ability to self-manage the goal activities long-term. INPH patients therefore probably need continuous encouragement and support to maintain reached goals. Patient-centred care with the approach to reach self-management is fundamental in the European physiotherapy guideline for Parkinson’s disease. The guideline emphasises education and repeated support from professionals as well as carer involvement to promote the patients’ behavioural changes [9]. We believe that these recommendations are applicable to iNPH patients.

The HIFE programme is developed and feasible for persons with dementia [16, 24]. Using the programme in nursing home residences, the attendance rate was about 75% [16, 25] and about 70% of all exercise sessions were performed at high intensity [25]. Motivation and co-existing medical conditions seemed not to influence the benefit from high-intensity exercise in persons with dementia [26]. We conducted our research in primary care with the intervention performed by local physiotherapists with the aim of reflecting a clinical routine. This may be one reason for the low adherence to exercise in this study.

Finally, in the research area of iNPH there are relatively few large prospective studies exploring the outcome after shunt surgery [27–29]. This study brings not only new information about the role of physical exercise in iNPH but also adds important knowledge about the outcome after a shunt operation. The iNPH scale is constructed to evaluate outcome after surgery as a total score [6] and its potential for use in a larger context is explored here. Our findings are in line with the results of the European iNPH multi-centre study with significant postoperative improvements in the total scale score as well as in all the separate domain scores [5].

## Limitations

Our study has several limitations. The most severe limitation is the large dropout rate causing low adherence to the intervention, which entails loss of statistical power. This limitation probably leads to failure to identify differences in favour of intervention, as indicated by the numerical differences seen between the groups for many of the outcome measures. Future studies should be larger and focus on better adherence to exercise intervention. The intervention was performed in clinical practice by physiotherapists with variable experience and conditions that may have caused differences in supervision. There were also logistical problems for the coordinators to initiate the intervention to start in time. Additionally, shunt complications and comorbidity influenced the ability to perform the intervention. Highly supervised physical exercise clinical trials under controlled conditions often generate a higher adherence rate and optimal performance but physical exercise interventions have to be applicable in clinical practice [30]. The dropout risk is important to be aware of when studying iNPH rehabilitation in the future.

The double interventions design may have influenced the results. Both groups increased clearly in all domain scores postoperatively and the surgical effect may have covered the smaller effect from the rehabilitation intervention. Another factor to consider is that the control group may have followed the advice to exercise more than we had expected and that the difference between the groups therefore became smaller and not significant. The magnitude of exercise in the control group was unknown to us.

The primary outcome measure can be questioned. The iNPH scale is developed to evaluate the most affected symptom domains in iNPH after shunt surgery and is not validated to evaluate rehabilitation effects. The HIFE intervention is based on functional exercises of gait and balance and the lack of effects in the neuropsychology and continence domains might have been expected. Aerobic exercise at moderate intensity has been shown to have a positive effect on cognition functions in patients with mild cognitive impairment [31]. However, the HIFE programme has not been shown to have beneficial effects on cognition in individuals with dementia [32]. The individual goals in the present study were set in the enrolment process, months before the surgery. This may have influenced the effect and may have given an advantage to the exercise group overseen by the supervising physiotherapists.

## Conclusions

An additional high-intensity exercise intervention did not influence the total iNPH scale scores after shunt surgery for patients with iNPH. The exercise group improved more in the balance domain scores at the long-term follow-up and reached their set goals more frequently. Our results indicate that physical exercise and goal setting can be effective for individuals with iNPH. Future studies are required to understand the effect of different interventions and the feasibility in clinical practice. Research is also required to illustrate whether there are sex differences in the iNPH condition as well as in aspects of rehabilitation.

## Supplementary Information

**Additional file 1: **Home exercises.

**Additional file 2: **INPH scale decription.

**Additional file 3: **Changes from baseline in primary and secondary iNPH scale scores for the ITT population.

**Additional file 4: **Changes from baseline in primary and secondary iNPH scale scores for the PP population.

## Data Availability

Anonymised source data are available on reasonable request from the corresponding author.

## Abbreviations

CI: Confidence interval
GAS: Goal attainment scaling
HIFE: High-intensity functional exercise
iNPH: Idiopathic normal pressure hydrocephalus
ITT: Intention-to-treat
MMSE: Mini mental state examination
PP: Per protocol
RCT: Randomized clinical trial
SD: Standard Deviation
