# Supplementary material for: Physical exercise and goal attainment after shunt surgery in idiopathic normal pressure hydrocephalus: a randomised clinical trial
Source: Fluids Barriers CNS. 2021 Nov 22;18:51. doi: 10.1186/s12987-021-00287-8 (PMC8607575; doi:10.1186/s12987-021-00287-8)
Supplement: Supplementary file 1 — Additional file 1: Home exercises. [file 12987_2021_287_MOESM1_ESM.pdf]

Exercise diary

Monday	Tuesday	Wednesday	Thursday	Friday	Saturday	Sunday

Normal pressure hydrocephalus

Physical activity before and after your shunt surgery

Normal pressure hydrocephalus is a condition with increased amount of liquid within the cavities of the brain. The condition has a variation of symptoms but you always have some kind of disturbed gait and/or balances.

The symptoms can occur in slow progression and you will often gradually change your behaviour to avoid activities that require good balance. Reduced physical activity leads to decreased cardiovascular fitness, strength and mobility.

Before your surgery, it is important to maintain or if possible increase your physical performance. After surgery, the need of physical activity remains and it is important to try to increase your activities in general.

The home exercises presented here are recommended daily five days per week.
It is often easier to remember the exercises if you exercise at the same time every day. At the end of this folder, you can find an exercise diary were you can note your exercise sessions.

In addition with the home exercises, it is important to maintain the walking ability and we recommend you to take daily walks based on your physical condition.

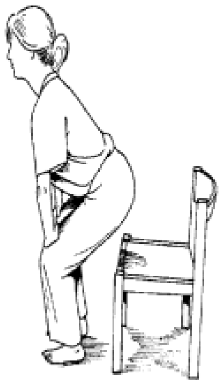

Keep your hands on the knees and keep your back in an upright position. Lean forward and rise partly. Sit down slowly.
Repeat 5 to 10 times in 2 sets.

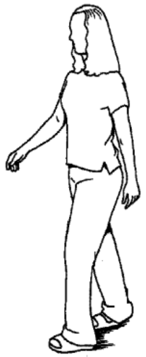

Walk a distance and count each step. Try to increase the step length and walk the same distance with fewer steps.
Repeat 10 times in 2 sets.

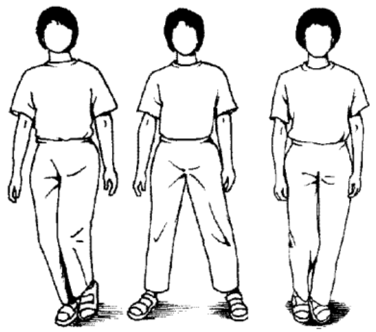

Take one step to the left and then one step to the right. Stand with your feet together between each step.
Repeat 5 to 10 times.

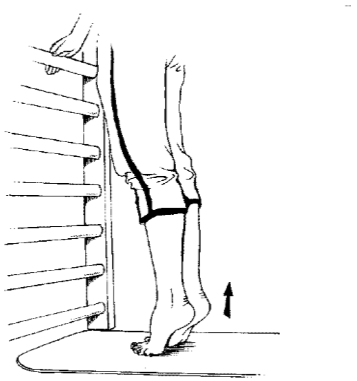

Calf raises. If possible, try to do the exercise on one leg.
Repeat 15 to 20 times.

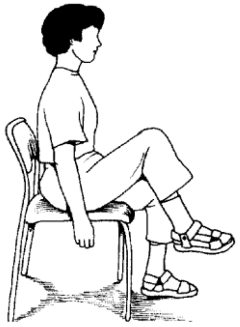

Raise your leg towards your stomach. Avoid contact with the backrest.
Repeat 10 times with each leg.

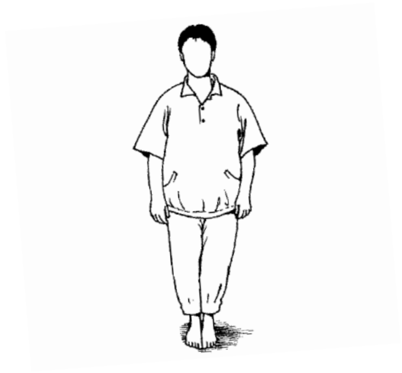

Stand in a corner with your feet together. If possible, close your eyes and count as far as you can.
Repeat 2 times.

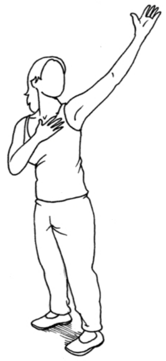

Stand with your feet apart. Lift your arm and turn your body looking at your hand. Repeat on the other side. Repeat 5 to 10 times.

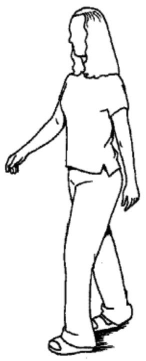

Walk at least 10 steps and turn your head to the left and to the right side.
Repeat.
