# Supplementary material for: Physical exercise and goal attainment after shunt surgery in idiopathic normal pressure hydrocephalus: a randomised clinical trial
Source: Fluids Barriers CNS. 2021 Nov 22;18:51. doi: 10.1186/s12987-021-00287-8 (PMC8607575; doi:10.1186/s12987-021-00287-8)
Supplement: Supplementary file 2 — Additional file 2: INPH scale decription. [file 12987_2021_287_MOESM2_ESM.pdf]

The iNPH scale

Gait domain

The rating from an ordinal gait scale and the results from the 10-metre walk test (number of steps and time in seconds, mean of two consecutive trails) are converted to the following scores from 0 to 100. A mean gait domain score is calculated from the available converted scores.

**Ordinal rating of gait**	**10 m walk test** (steps)	**10 m walk test** (seconds)
1. Normal gait = 100	<15.50 = 100	<8.75 = 100
2. Slight disturbance of tandem walk and turning = 86	15.50–16.50 = 90	8.75–9.25 = 90
	16.75–17.25 = 80	9.50–9.75 = 80
3. Wide-based gait with sway, without foot corrections = 71	17.50–18.00 = 70	10.00–10.25 = 70
	18.25–19.25 = 60	10.50–10.75 = 60
4. Tendency to fall, with foot corrections = 57	19.50–20.25 = 50	11.00–11.50 = 50
5. Walking with cane = 43	20.50–21.25 = 40	11.75–13.00 = 40
6. Bi-manual support needed = 29	21.50–23.75 = 30	13.25–16.00 = 30
7. Aided = 14	24.00–27.25 = 20	16.25–19.25 = 20
8. Wheelchair bound = 0	27.50–40.00 = 10	19.50–27.00 = 10
	>40 or fail = 0	>27 or fail = 0

Balance domain

The ordinal balance scale score is converted to the following balance domain scores from 0 to 100.

**Ordinal rating of balance**
1. Able to stand independently for more than 30 s on either lower extremity alone = 100
2. Able to stand independently for 5 - 30 s on either lower extremity alone = 83
3. Able to stand independently with the feet together (at the heels) for more than 30 s = 67
4. Able to stand independently with the feet together for 5 - 30 s = 50
5. Able to stand independently with the feet apart (one foot length) for more than 30 s = 33
6. Able to stand independently with the feet apart for 5 - 30 s = 17
7. Unable to stand without assistance = 0

Neuropsychology domain

The results from four separate tests are converted to the following scores from 0 to 100. A mean neuropsychology domain score is calculated from the available converted scores.

**Grooved pegboard** (fastest trial)	**Rey Auditory Verbal Learning Test** (sum of five trials)	**Stroop, colour-naming**	**Stroop, interference**
<79 = 100	>44 = 100	<68 = 100	<132 = 100
79–87 = 90	38–44 = 90	68–77 = 90	132–160 = 90
88–96 = 80	32–37 = 80	78–81 = 80	161–188 = 80
97–105 = 70	30–31 = 70	82–87 = 70	189–210 = 70
106–114 = 60	28–29 = 60	88–96 = 60	211–239 = 60
115–128 = 50	26–27 = 50	97–106 = 50	240–298 = 50
129–144 = 40	22–25 = 40	107–121 = 40	299–385 = 40
145–173 = 30	19–21 = 30	122–134 = 30	386–600 = 30
174–245 = 20	15–18 = 20	135–171 = 20	>600 = 20
246–600 = 10	11–14 = 10	172–300 = 10	Fail = 10
>600 or fail = 0	<11 or fail = 0	>300 or fail = 0	

Continence domain

The ordinal continence scale score is converted to the following continence domain scores from 0 to 100.

**Ordinal rating of continence**
1 Normal = 100
2 Urgency without incontinence = 80
3 Infrequent (not daily) incontinence without napkin = 60
4 Frequent incontinence with napkin = 40
5 Bladder incontinence (i.e. complete loss of bladder control) = 20
6 Bladder and bowel incontinence (i.e. complete loss of bladder and bowel control) = 0

Total iNPH score

The total iNPH scale score is the mean of the available domain scores with the gait domain score x 2.

Total iNPH score
2 x Gait domain + Balance domain + Neuropsychology domain + Continence domain<br>5 (or number of available domain scores)

Reference

Hellström P, Klinge P, Tans J, Wikkelsø C. A new scale for assessment of severity and outcome in iNPH. Acta Neurol Scand. 2012 May 16. doi:10.1111/j.1600-0404.2012.01677.x.
