# Supplementary material for: Physical exercise and goal attainment after shunt surgery in idiopathic normal pressure hydrocephalus: a randomised clinical trial
Source: Fluids Barriers CNS. 2021 Nov 22;18:51. doi: 10.1186/s12987-021-00287-8 (PMC8607575; doi:10.1186/s12987-021-00287-8)
Supplement: Supplementary file 3 — Additional file 3: Changes from baseline in primary and secondary iNPH scale scores for the ITT population. [file 12987_2021_287_MOESM3_ESM.pdf]

Changes from baseline in primary and secondary iNPH scale scores for the ITT population

	Exercise group (n = 50)	*p*-value within group	Control group (n = 59)	*p*-value within group	*p*-value between groups	Difference between groups Mean (95% CI)
**Primary outcome**						
Total iNPH scale score (0-100)						
Baseline<br>Mean (SD)<br>Median (min;max)	58.7 (15.3)<br>60.5 (27.2; 91.1)<br>50		55.4 (16.3)<br>52.9 (22; 89.6)<br>59		0.28	3.27 (-2.72; 9.34)
Post-intervention	74.5 (15.9)<br>78.1 (27.3; 97.5)<br>n = 50		70.5 (15.9)<br>72.7 (34.2; 100)<br>n = 58		0.19	4.05 (-2.07; 10.14)
Change from baseline	15.9 (13.2)<br>14.2 (-21.4; 48.4)<br>n = 50	**<.0001**	14.9 (12.2)<br>13.4 (-11.5; 53.3)<br>n = 58	**<.0001**		0.94 (-3.86; 5.84)
6-month follow-up	77.8 (14.4)<br>81.2 (47.2; 99.5)<br>n = 43		73.4 (17.1)<br>77.9 (35.2; 99.5)<br>n=n = 52		0.18	4.38 (-2.12; 10.84)
Change from baseline	19.1 (12.5)<br>16.2 (-3.3; 49.4)<br>n = 43	**<.0001**	17.0 (12.5)<br>15.4 (-15.7; 41.1)<br>n = 52	**<.0001**	0.41	2.09 (-3.05; 7.14)
**Secondary outcomes**						
Gait iNPH scale score (0-100)						
Baseline	53.3 (22.9)<br>50.5 (9.7; 100)<br>n = 50		50.9 (23.7)<br>43.7 (9.7; 100)<br>n = 59		0.59	2.37 (-6.49; 11.22)
Post-intervention	74.9 (23.0)<br>82 (19.7; 100)<br>n = 50		70.1 (26.0)<br>72.8 (16.3; 100)<br>n = 58		0.31	4.78 (-4.55; 14.21)
Change from baseline	21.6 (17.3)<br>21.5 (-36; 66.3)<br>n = 50	**<.0001**	18.8 (19.4)<br>17 (-18.3; 60.7)<br>n = 58	**<.0001**	0.44	2.76 (-4.25; 9.83)
6-month follow-up	79.8 (21.7)<br>85.3 (25.7; 100)<br>n = 43		73.4 (25.9)<br>82 (16.3; 100)<br>n = 52		0.20	6.39 (-3.41; 16.20)
Change from baseline	26.9 (17.2)<br>27.3 (-0.7; 76.3)<br>n = 43	**<.0001**	21.6 (20.6)<br>18.2 (-30; 69.7)<br>n = 52	**<.0001**	0.18	5.32 (-2.50; 13.07)
Balance iNPH scale score (0-100)						
Baseline	68.8 (14.1)<br>67 (33; 100)<br>n = 50		67.0 (15.9)<br>67 (17; 100)<br>n = 59		0.56	1.73 (-4.00; 7.48)
Post-intervention	76.7 (11.5)<br>83 (50; 100)<br>n = 50		74.5 (10.2)<br>67 (50; 100)<br>n = 58		0.32	2.20 (-1.92; 6.32)
Change from baseline	7.92 (16.14)<br>0 (-17; 67)<br>n = 50	**0.0006**	7.72 (14.96)<br>0 (-17; 50)<br>n = 58	**0.0004**	0.98	0.196 (-5.731; 6.045)
6-month follow-up	80.9 (11.1)<br>83 (67; 100)<br>n = 43		74.7 (11.9)<br>83 (33; 100)<br>n = 52		**0.0081**	6.28 (1.52; 11.00)
Change from baseline	11.9 (14.2)<br>16 (0; 67)<br>n = 43	**<.0001**	5.96 (12.69)<br>0 (-34; 34)<br>n = 52	**0.0025**	**0.039**	5.90 (0.54; 11.28)

Neuropsychology iNPH scale score (0-100)						
Baseline	57.0 (18.6)<br>57.5 (20; 87.5)<br>n = 50		55.0 (19.2)<br>52.5 (17.5; 95)<br>n = 59		0.61	1.91 (-5.36; 9.09)
Post-intervention	67.1 (16.8)<br>68.8 (25; 95)<br>n = 50		62.8 (19.6)<br>65 (12.5; 100)<br>n = 57		0.24	4.29 (-2.81; 11.39)
Change from baseline	10.2 (11.2)<br>8.8 (-25; 32.5)<br>n = 50	**<.0001**	8.16 (12.31)<br>7.5 (-30; 45)<br>n = 57	**<.0001**	0.40	1.99 (-2.50; 6.54)
6-month follow-up	68.5 (18.2)<br>70 (25; 97.5)<br>n = 43		66.9 (20.7)<br>70 (17.5; 100)<br>n = 51		0.69	1.63 (-6.36; 9.69)
Change from baseline	11.6 (11.1)<br>12.5 (-15; 37.5)<br>n = 43	**<.0001**	10.4 (11.0)<br>7.5 (-20; 35)<br>n = 51	**<.0001**	0.62	1.19 (-3.37; 5.68)
Continence iNPH scale score (0-100)						
Baseline	61.2 (28.7)<br>60 (0; 100)<br>n = 50		53.2 (30.3)<br>40 (0; 100)<br>n = 59		0.18	7.98 (-3.48; 19.29)
Post-intervention	79.2 (28.6)<br>90 (0; 100)<br>n = 50		74.3 (26.6)<br>80 (0; 100)<br>n = 56		0.39	4.91 (-5.71; 15.71)
Change from baseline	18.0 (29.8)<br>10 (-40; 100)<br>n = 50	**<.0001**	21.1 (28.4)<br>10 (-20; 100)<br>n = 56	**<.0001**	0.64	-3.07 (-14.29; 8.00)
6-month follow-up	80.0 (23.1)<br>80 (0; 100)<br>n = 43		78.5 (27.7)<br>100 (0; 100)<br>n = 52		0.83	1.54 (-9.00; 12.17)
Change from baseline	18.1 (26.8)<br>20 (-40; 100)<br>n = 43	**<.0001**	25.0 (28.0)<br>20 (-20; 100)<br>n = 52	**<.0001**	0.25	-6.86 (-18.10; 4.35)

Changes from baseline for primary and secondary outcomes in iNPH scale scores at the post-intervention follow-up and the long-term follow-up six months postoperatively. ***ITT*** intention-to-treat. Bold values indicate significance of p ≤ 0.05. Values are presented as mean (SD), median (min; max) and the difference between groups as mean difference and 95% CI.
