# Supplementary material for: Physical exercise and goal attainment after shunt surgery in idiopathic normal pressure hydrocephalus: a randomised clinical trial
Source: Fluids Barriers CNS. 2021 Nov 22;18:51. doi: 10.1186/s12987-021-00287-8 (PMC8607575; doi:10.1186/s12987-021-00287-8)
Supplement: Supplementary file 4 — Additional file 4: Changes from baseline in primary and secondary iNPH scale scores for the PP population. [file 12987_2021_287_MOESM4_ESM.pdf]

Changes from baseline in primary and secondary iNPH scale scores for the PP population

	Exercise group (n=28)	*p*-value within group	Control-group (n=58)	*p* value within group	*p*-value between groups	Difference between groups Mean (95% CI)
**Primary outcome**						
Total iNPH scale score (0-100)						
Baseline<br>Mean (SD)<br>Median (min;max)	56.8 (15.7)<br>58.6 (27.2; 88.3)<br>n = 28		55.6 (16.4)<br>53.1 (22; 89.6)<br>n = 58		0.75	1.25 (-6.12; 8.69)
Post-intervention	76.7 (14.7)<br>80.5 (37.2; 97.5)<br>n = 28		70.5 (15.9)<br>72.7 (34.2; 100)<br>n = 58		0.081	6.21 (-0.83; 13.45)
Change from baseline	19.9 (12.9)<br>16.8 (-0.5; 48.4)<br>n = 28	**<.0001**	14.9 (12.2)<br>13.4 (-11.5; 53.3)<br>n = 58	**<.0001**	0.094	4.96 (-0.86; 10.59)
6-month follow-up	77.1 (15.9)<br>79.7 (47.2; 99.5)<br>n = 27		73.3 (17.3)<br>78.2 (35.2; 99.5)<br>n = 51		0.35	3.74 (-4.09; 11.61)
Change from baseline	20.6 (14.1)<br>16.7 (-3.3; 49.4)<br>n = 27	**<.0001**	16.7 (12.5)<br>15.2 (-15.7; 41.1)<br>n = 51	**<.0001**	0.21	3.90 (-2.22; 9.98)
**Secondary outcomes**						
Gait iNPH scale score (0-100)						
Baseline	51.3 (24.3)<br>48.7 (9.7; 95.3)<br>n = 28		51.2 (23.8)<br>43.7 (9.7; 100)<br>n = 58		0.59	2.37 (-6.49; 11.22)
Post-intervention	76.8 (21.1)<br>82 (23; 100)<br>n = 28		70.1 (26.0)<br>72.8 (16.3; 100)<br>n = 58		0.23	6.71 (-4.28; 17.95)
Change from baseline	25.4 (17.1)<br>25.8 (-8.3; 66.3)<br>n = 28	**<.0001**	18.8 (19.4)<br>17 (-18.3; 60.7)<br>n = 58	**<.0001**	0.13	6.61 (-2.02; 15.19)
6-month follow-up	80.1 (22.6)<br>87 (25.7; 100)<br>n = 27		72.9 (25.9)<br>82 (16.3; 100)<br>n = 51		0.21	7.27 (-4.31; 19.18)
Change from baseline	28.7 (19.9)<br>29.7 (-0.7; 76.3)<br>n = 27	**<.0001**	20.6 (19.6)<br>18 (-30; 57.7)<br>n = 51	**<.0001**	0.092	8.05 (-1.33; 17.46)
Balance iNPH scale score (0-100)						
Baseline	66.1 (16.0)<br>67 (33; 100)<br>n = 28		66.8 (15.9)<br>67 (17; 100)<br>n = 58		0.56	1.73 (-4.00; 7.48)
Post-intervention	78.0 (12.7)<br>83 (50; 100)<br>n = 28		74.5 (10.2)<br>67 (50; 100)<br>n = 58		0.21	3.48 (-1.78; 8.57)
Change from baseline	11.8 (19.1)<br>16 (-17; 67)<br>n = 28	**0.0029**	7.72 (14.96)<br>0 (-17; 50)<br>n = 58	**0.0004**	0.31	4.10 (-3.61; 11.37)
6-month follow-up	81.4 (12.4)<br>83 (67; 100)<br>n = 27		74.5 (12.0)<br>83 (33; 100)<br>n = 51		**0.015**	6.95 (1.13; 12.71)
Change from baseline	15.3 (16.0)<br>16 (0; 67)<br>n = 27	**<.0001**	6.08 (12.79)<br>0 (-34; 34)<br>n = 51	**0.0027**	**0.0062**	9.25 (2.52; 15.74)
Neuropsychology iNPH scale score (0-100)						

Baseline	57.4 (19.9)<br>56.3 (20; 87.5)<br>n = 28		55.2 (19.3)<br>52.5 (17.5; 95)<br>n = 58		0.61	1.91 (-5.36; 9.09)
Post-intervention	69.1 (17.5)<br>71.3 (25; 95)<br>n = 28		62.8 (19.6)<br>65 (12.5; 100)<br>n = 57		0.16	6.30 (-2.24; 15.00)
Change from baseline	11.7 (9.2)<br>10 (-2.5; 32.5)<br>n = 28	**<.0001**	8.16 (12.31)<br>7.5 (-30; 45)<br>n = 57	**<.0001**	0.19	3.54 (-1.71; 8.68)
6-month follow-up	66.7 (19.1)<br>65 (25; 97.5)<br>n = 27		67.0 (20.9)<br>72.5 (17.5; 100)<br>n = 50		0.96	-0.283 (-9.833; 9.375)
Change from baseline	10.4 (11.8)<br>12.5 (-15; 37.5)<br>n = 27	**<.0001**	10.3 (11.1)<br>7.5 (-20; 35)<br>n = 50	**<.0001**	1.00	0.070 (-5.294; 5.385)
Continence iNPH scale score (0-100)						
Baseline	57.9 (25.7)<br>60 (0; 100)<br>n = 28		53.4 (30.5)<br>40 (0; 100)<br>n = 58		0.18	7.98 (-3.48; 19.29)
Post-intervention	82.9 (25.9)<br>100 (0; 100)<br>n = 28		74.3 (26.6)<br>80 (0; 100)<br>n = 56		0.20	8.57 (-3.33; 21.11)
Change from baseline	25.0 (28.6)<br>20 (-40; 100)<br>n = 28	**0.0002**	21.1 (28.4)<br>10 (-20; 100)<br>n = 56	**<.0001**	0.59	3.93 (-9.47; 16.84)
6-month follow-up	77.0 (26.4)<br>80 (0; 100)<br>n = 27		79.2 (27.4)<br>100 (0; 100)<br>n = 51		0.80	-2.18 (-14.74; 11.00)
Change from baseline	20.0 (28.3)<br>20 (-40; 100)<br>n = 27	**0.0012**	25.5 (28.0)<br>20 (-20; 100)<br>n = 51	**<.0001**	0.46	-5.49 (-18.82; 7.50)

Changes from baseline for primary and secondary outcomes in iNPH scale scores at the post-intervention follow-up and the long-term follow-up six months postoperatively. ***PP*** Per protocol. Bold values indicate significance of $p \leq 0.05$. Values are presented as mean (SD), median (min; max) and the difference between groups as mean difference and 95% CI.
